# Efficacy of colchicine in oral submucous fibrosis: A systematic review and meta-analysis

**DOI:** 10.6026/973206300211087

**Published:** 2025-05-31

**Authors:** Ajay Kumar, Sonam Sah, Neha Sah, Aruna Das, Monika Singh, Praveen Kumar Singh, Sneha Gupta, Peeyush Shivhare, Prabhakar Kumar, Amit Kumar Bansa

**Affiliations:** 1 Department of Oral Medicine and Radiology, Institute of Medical Sciences, Banaras Hindu University, Varanasi-221005, Uttar Pradesh, India; 2 Department of Dentistry, All India Institute of Medical Sciences, Gorakhpur, Kunraghat-273008, India; 3 Department of Dentistry, MaaVindhyavasini Autonomous State Medical College, Mirzapur, Uttar Pradesh, India; 4 Department of Oral Medicine & Radiology, Dental College Azamgarh, Azamgarh, Uttar Pradesh, India; 5 Department of Oral and Maxillofacial Surgery, Institute of Sardar Patel Post Graduate Institute of Dental and Medical Sciences, Lucknow, India; 6 Department of Dentistry, Baba Kinaram Autonomous State Medical College, Chandauli, Uttar Pradesh, India; 7 Department of Oral Medicine and Radiology, Guru Nanakdev Dental College and Research Institute, Sunam, Punjab, India

**Keywords:** Colchicine, oral sub-mucous fibrosis, potentially malignant disorder

## Abstract

One of the potentially malignant disorders of the oral mucosa in Asian nations is known as oral submucous fibrosis (OSMF). The drug, surgical and combination therapies are some of the several OSMF treatment options. The importance of colchicine in the treatment of oral submucous fibrosis is still unclear. Therefore, it is of interest to review the efficacy of colchicine in oral submucous fibrosis in terms of improving symptoms such as burning sensation and mouth opening. Known data shows colchicine is in alleviating these symptoms in OSMF patients.

## Background:

The Latinized Greek name for cancer is "Karkinos," which means crab. This describes how a carcinoma invades nearby tissues, much like a crab's claws. The oral mucosa experiences clinical alterations before the majority of oral malignancies (mostly oral squamous cell carcinomas), which often manifest as a white or red patch and validate the two-step cancer development process [1, 2]. Precancer, pre-neoplastic, premalignant, epithelial precursor, carcinoma-prone, intraepithelial carcinoma, intraepithelial neoplasia, and potentially malignant disorders are some of the terms used to describe these early alterations. The term "oral potentially malignant disease" (OPMD), which is defined as "any oral mucosal abnormality that is linked with a statistically increased risk of developing oral cancer," was proposed by the World Health Organization (WHO) in 2007 for such lesions in the oral cavity [3]. The conditions that can cause OPMD include candidal leukoplakia, proliferative verrucous oral submucous fibrosis, actinic cheilitis, oral lichen planus, oral lichenoid lesions, oral lupus erythematosus, dyskeratosiscongenita, palatal lesions in reverse smoking, erythroplakia, and leukoplakia [4]. One of the most common OPMDs seen in clinics is oral submucous fibrosis, especially in Asian countries. OSMF was defined as an "insidious, chronic disease affecting any part of the oral cavity and sometimes the pharynx. Although occasionally preceded by and/or associated with juxtaepithelial inflammatory reaction followed by a fibroelastic change of the lamina propria with epithelial atrophy leading to stiffness of mucosa and causing an inability to eat" [5]. The most significant suspected etiologic agent for this entity is areca nut. Alkaloids and flavonoids included in areca nuts eventually boost collagen production and reduce collagen breakdown through a separate method. In the end, it causes fibrosis of the oral cavity, whether or not the pharynx is present [6]. This condition clinically presents with blanching of the oral mucosa, burning sensation, fibrous bands within the mucosa, shrunken uvula, reduced mouth opening, restricted tongue movements, depapillation of the tongue, and difficulty in chewing food and vesicles [7]. The various treatment modalities of OSMF include pharmacological, surgical, and combined therapy. Various pharmacological agents proposed to be the management of OSMF are steroids, hyaluronidase, collagenase, placental extract, levamisole, pentoxifylline, curcumin, and antioxidants [8, 9]. Colchicum autumnale, a crocus-like plant named after the Colchis region near the eastern edge of the Black Sea, is the natural source of the alkaloid colchicine. Colchicine is a long-standing drug known for its anti-inflammatory and anti-fibrotic properties. Numerous in vitro and in vivo studies have thoroughly demonstrated its pharmacodynamic effects as an anti-fibrotic agent, providing strong evidence for its potential use in treating various fibrosis-related conditions [10]. Colchicine has been shown in the literature to have beneficial effects in reducing mouth opening and burning sensation in oral submucous fibrosis [11]. Therefore, it is of interest to review the efficacy of colchicine in oral submucous fibrosis in terms of improving symptoms such as burning sensation and mouth opening.

## Methods:

## Protocol and registration:

The PRISMA checklist for performing systematic review and meta-analysis was used in this investigation [16]. The review was registered in PROSPERO with registration number CRD42022379193.

## Eligibility criteria:

## Inclusion criteria / selection criteria:

The "PICOS" framework was utilized to narrow down the study question. The inclusion and exclusion criteria were determined using the research question [17].

[1] Population (P) - Individuals in any age group diagnosed clinically and/or histopathologically with Oral Submucous Fibrosis.

[2] Intervention (I) - Colchicine in any form

[3] Comparison (C) - The comparison group consisted of OSMF with any other drug modality and/or standard treatment.

[4] Outcome (O) - The major outcomes examined were burning sensation and mouth opening, with secondary outcomes of cheek flexibility being taken into account if applicable.

## Exclusion criteria:

Publications written in languages other than English as well as editorials, case reports, comments, and articles on animals were omitted. The search was carried out impartially by two researchers and the outcomes of the repeated searches were excluded.

## Search Strategy / Data Source:

The literature search was conducted in PubMed, Scopus, Web of Science, Embase, Ovid, Google Scholar, and the Cochrane Database / Cochrane Central using selected keywords with the Boolean operators "AND" and "OR" based on the study objectives. Moreover, a manual search of the literature and the reference list of selected articles were undertaken. Using electronic and other search techniques that were obtained in their full, we checked all pertinent items that were discovered. Searching conference proceedings and abstracts turned up unpublished research. Colchicine in the treatment of oral submucous fibrosis, oral submucous fibrosis, therapy of oral submucous fibrosis, and potentially malignant disorders were some of the keywords used to search databases.

## Risk of bias assessment:

With the help of the Cochrane Handbook for Systematic Reviews of Interventions, the caliber of the studies that were obtained was evaluated [18].

## Statistical analysis and Meta-analysis:

RevMan 5.4 was used to apply the random-effects model to the meta-analyses (RevMan 5.4, The Nordic Cochrane Centre and Copenhagen).

## Results:

A total of 41 articles were retrieved from the databases and after the removal of duplicates and automation tools, 30 articles were screened for title and abstract, of which 15 were excluded. Four articles could not be retrieved. Out of 11 articles, 6 were excluded due to various reasons. A total of 5 articles were used for qualitative analysis, whereas 2 (Neupane *et al.* 2016, Mounika *et al.* 2021, [12, 15] were used for quantitative analysis/meta-analysis. The other 3 articles (Krishnamoorthy *et al.* Daga *et al.* 2017, Sharma *et al.* 2020, [11, 12, 13-14] were excluded from meta-analysis due to incomplete information regarding statistical analysis and data. The study search process conducted according to the PRISMA guidelines is shown in Figure 1 - (see PDF).

## General characteristics of the included studies:

Table 1 provides summaries of the study characteristics. It summarizes the various variables in the study such as number of participants, whether histopathologically confirmed, intervention details, age, gender, staging/grouping ollowed, duration of the study, follow-up periods, details of variables and outcome.

## Quality assessment and evidence assessment / risk of bias assessment:

With the help of the Cochrane Handbook for Systematic Reviews of Interventions, the caliber of the studies that were obtained was evaluated [18]. The criteria that were looked into included the creation of random sequences (selection bias), hiding allocations (selection bias), blinding participants and employees (performance bias), blinding outcome assessments (detection bias), having insufficient outcome data (attrition bias), and selective reporting (reporting bias). Mounika *et al.* 2021 [15] reported an overall low risk of bias; however, the other studies' risk assessments were often high due to subpar research design and higher risks of performance, detection, attrition, and reporting bias (Table 2). To ensure high-quality investigations, this necessitates multi-centered clinical trial analysis created with specific criteria and established technique.

## Meta-analysis:

For meta-analysis, the study performed by Daga *et al.* 2017 [13] was excluded due to the unavailability of complete data statistics and both the treatment groups had colchicine, while the studies of Krishnamoorthy *et al.* 2013 [11] and Sharma *et al.* 2020 [14] were excluded due to the unmatched drug of the control group and outcome not reported in mean and standard deviation format. A total of 2 studies were included in the meta-analysis. The Q test was used to evaluate heterogeneity and I^2^ statistics were used to quantify it. Selected studies' mean and standard deviation data were gathered. The main outcomes were determined by the mean mouth opening and mean burning sensation scores. Two separate comparisons were performed: Comparisons of mouth opening between Colchicine & dexamethasone plus hyaluronidase, and comparisons of burning sensation between Colchicine & dexamethasone plus hyaluronidase using standardized mean difference (MD) for mouth opening and burning sensation. If the test revealed significant heterogeneity (I^2^ > 50%), a random-effects model was employed for the analyses; otherwise (I^2^ ≤ 50%), a fixed-effects model was utilized.

## Mouth opening:

Improvement in mouth opening was reported in both studies, with 35 subjects in the colchicine group and 35 in the dexamethasone plus hyaluronidase group. A significant improvement in mouth opening (measured in mm) was observed in the dexamethasone plus hyaluronidase group compared to the colchicine group, with a mean difference of 1.12 (confidence interval [CI] = 0.34 to 2.09; P < 0.05; Z = 2.72) from the treatment initiation to end of the treatment (Figure 2 - see PDF).

## Burning sensation:

Improvement in burning sensation was reported in both studies, with 35 subjects in the colchicine group and 35 in the dexamethasone plus hyaluronidase group. Compared to the colchicine group, the dexamethasone plus hyaluronidase group showed significant reduction of burning sensation (measured in VAS scale), with a mean difference of 0.54 (confidence interval [CI] = 0.06 to 1.03; P < 0.05; Z = 2.19) from the treatment initiation to end of the treatment (Figure 3 - see PDF).

## Discussion:

Oral submucous fibrosis (OSMF) is a chronic, potentially malignant condition predominantly affecting the oral cavity and oropharynx. Although it has been reported among Indian-origin populations in South Africa and certain parts of the United States, its prevalence is notably higher in South Asian countries such as India, Sri Lanka, Pakistan, China, and Bangladesh [19]. OSMF most commonly affects individuals between 20 and 40 years of age and is characterized by progressive fibrosis leading to restricted mouth opening, difficulty in chewing, swallowing, and speaking, often accompanied by a burning sensation due to epithelial atrophy [20]. The premalignant nature of OSMF was first described by Paymaster in 1956 [21], and since then, malignant transformation rates (MTRs) ranging from 0.9% to 26.6% have been reported [22, 23]. A recent meta-analysis found an overall MTR of approximately 6%, reinforcing the importance of early diagnosis and intervention to improve outcomes and reduce the risk of malignant progression [24]. Numerous treatment modalities have been investigated over the years [8]. Intralesional corticosteroids such as dexamethasone, hydrocortisone, or triamcinolone, often combined with hyaluronidase, remain standard therapy. However, their effectiveness in advanced cases is limited, prompting the exploration of alternative therapies with better safety profiles and efficacy [7]. Colchicine, known for its anti-inflammatory and anti-fibrotic properties, has been studied in the context of OSMF. Its pharmacological effects are mediated by multiple mechanisms: it disrupts microtubule formation, inhibiting collagen synthesis; enhances collagen degradation; impairs intracellular collagen transport in fibroblasts; and down regulates fibrogenic cytokines like TGF-β, IL-4, and IL-6 [13]. Colchicine has demonstrated effectiveness in various fibrotic disorders and is generally well tolerated at moderate doses. Common side effects such as nausea, vomiting, and abdominal discomforts are typically dose-dependent and reversible [26]. This review included studies exclusively evaluating colchicine monotherapy in OSMF to reduce heterogeneity. However, some variability remained due to differing diagnostic methods and non-standardized outcome assessment across studies. The most commonly assessed outcomes were mouth opening and burning sensation. Burning sensation improved in all studies. Statistically significant improvement was noted in studies by Neupane *et al.* although some studies did not report statistical data or used non-standardized tools [12]. The Visual Analogue Scale (VAS), used in several studies, offered a reliable method for quantifying pain and discomfort. The reduction in burning sensation is likely attributable to colchicine's anti-inflammatory mechanism. Mouth opening also showed marked improvement across most studies. Mounika *et al.* [15] reported highly significant improvement (p < 0.001), while others like Sharma *et al.* reported statistically significant outcomes (p < 0.05) [14]. However, Daga *et al.* did not include statistical analysis [13]. Mounika *et al.* also assessed additional clinical parameters like cheek flexibility and tongue protrusion, which improved with colchicine but to a lesser extent than in control groups, and the intergroup differences were not statistically significant [15]. Histological outcomes were considered in a few studies but were not uniformly reported. While colchicine shows promise as a treatment option for OSMF, there is a need for further high-quality studies with larger sample sizes, standardized diagnostic protocols, and validated outcome measures. These are essential for generating robust, generalizable evidence. A recent systematic review by Rai *et al.* also supported colchicine's therapeutic potential while emphasizing the need for further research in this area [27].

## Conclusion:

Known data shows colchicine is in alleviating these symptoms in OSMF patients.

## Financial support and sponsorship:

Nil

## Figures and Tables

**Table 1:** Showing details of population, study groups and outcome (PICO) of included studies

**Author**	**Study groups (No of participants)**	**H/P done**	**Intervention preparation/ Dose**	**Age mean range**	**Gender**	**Grouping**	**Duration (months)**	**Follow-up months**	**Variables evaluated**	**Outcome**
Mounika *et al.* 2021	30 patients with OSMF Group A 15 patient Group B 15 patient	NO	Group A 0.5 mg Colchicine tablets twice daily for 3 months	42.65 ± 11.51 (Mean ± SD)	Group A 11 males, 4 females.	OSMF of stages I, II, III.	Group A 3 months.	3 and 6 months.	Burning sensation, Inter- incisal mouth opening, Buccal mucosal flexibility, Tongue protrusion	Group A Showed significant improvement in burning sensation and mouth opening.
			Group B Intralesional injection of dexamethasone 2 mL, Hyaluronidase 1,500 IU with 2 mL lignocaine HCl biweekly for 4-6 weeks.		Group B 10 males, 5 females		Group B 4 to 6 weeks.			Group B Showed significant improvement in mouth opening and tongue protrusion.
Sharma *et al.* 2021	50 Patients	Punch Biopsy	Group A 0.5 mg Colchicine tablets twice daily Hyaluronidase 1,500 IU 1 ml of lignocaine. 0.5ml	25 (50%) 20-30 years. 15 (30%) patients 30-40 years, 10 -20% patients 40-50 years	50 patients	OSMF based on the WHO criteria	Both Groups	Not mentioned	Ulceration, Xerostomia, Burning sensation, Limited Open Mouth	Group 1 Showed significant improvement in burning sensation and mouth opening
	40 males 10 females	Pre & Post Treatment	Group B Hyaluronidase 1,500 IU and 0.5 ml of injection Hydrocortisone acetate 25 mg/ml		40 males 10 females	Stage III Stage IV Stage V	16 Weeks			Group 2 Showed significant improvement in mouth opening and mouth opening
Daga *et al.* 2017	30 patients with OSMF	NO	Group A 0.5 mg colchicines tablets twice daily plus intralesional hyaluronidase 1500IU plus 0.5 ml lignocaine hydrochloride at week interval for 12 week.	90% were male and young adults. (27:3)	Group A 15 patients	OSMF of grade II	Group A 12 weeks	6 months.	Limited mouth Opening	Group A maximum increase of about 8 mm.
	Group B 15 patient		Group B 0.5 mg colchicines tablets twice daily with intralesional injection of Triamcinolone Acetonide 10 mg/ml at weekly interval for 12 weeks.		Group B 15 patients		Group B 12 weeks		Burning sensation in oral cavity	Group B maximum increase of about 5 mm
Neupane *et al.* 2016	40 patients With OSMF	NO	Group A 0.5 mg colchicines tablets twice daily for 12 week.	Total mean age of 29.50 ±5.27 years		OSMF		Monthly follow up visits	Reduced mouth opening	Group A Mouth opening 25.20±5.88mm
	Group A 20 patient		Group B Injections Dexamethasone (4mg/ml) plus Hyaluronidase 1500 IU for Biweekly intralesional 12 week.	Group A 29.60 ± 6.78 years	Group A 20 Patients		Group A 12 weeks			Burning sensation 5.18±1. 38 score (VAS)
	Group B 20 patient			Group B 29.40 ± 3.31. years (Mean ± SD)	Group B20 Patients		Group B 12 weeks			Group B Mouth opening 29.75 ± 5.76mm Burning sensation 13.99±2.35 scores(VAS)
Krishnamoorthy and Khan *et al.* 2013	50 patients With OSMF	Punch Biopsy	Group 1 0.5 mg colchicine tablet twice daily and 0.5 ml Hyaluronidase 1,500 IU once a week for intralesional 12 week.	28.40 ± 8.6 (Mean ± SD)	Group A 25 Patients	OSMF Clinically graded according to Gupta CD *et al.*	Group A 12 weeks	Not mentioned	Burning sensation, Trismus, Intolerance to hot and spicy foods	All the patients in both the groups were relieved from burning sensation, Increase mouth opening
	Group A 25 patient	Pre & Post Treatment	Group 2 0.5 ml Hyaluronidase 1,500 IU and 0.5ml Hydrocortisone acetate 25 mg/ml for intralesional 12 week.		Group B 25 Patients		Group B 12 weeks			
	Group B 25 patient									

**Table 2:** Risk of bias assessment of all studies

	**Study Random sequence generation (Selection bias)**	**Allocation concealment (Selection bias)**	**Blinding of participants and personnel (performance bias)**	**Blinding of outcome assessment (detection bias)**	**Incomplet e outcome data (attrition bias)**	**Selective reporting (reporting bias)**
Mounika *et al.* 2021	+	+	-	-	+	+
Sharma *et al.* 2020	?	-	-	-	+	+
Daga *et al.* 2017	-	-	-	-	+	+
Neupane *et al.* 2016	?	-	-	-	+	+
Krishnamoorthy *et al.* 2013	?	-	-	-	+	+
